# A MRI-based open source tool for quantitative measurement of relaxation times and perfusion in cardiac tissues

**DOI:** 10.1186/1532-429X-18-S1-W33

**Published:** 2016-01-27

**Authors:** Ehsan Yazdanparast, Jesús María, Ruiz-Cabello Osuna, Ignacio Rodríguez, Ramirez de Arellano

**Affiliations:** Advanced Imaging, National Center of Cardiovascular Investigations(CNIC), Madrid, Spain

## Background

In cardiac Magnetic Resonance Imaging(MRI) studies, evaluating T_1_, T_2_ and T^*^_2_ relaxation times and hemodynamics assessment using perfusion analysis has an undeniable importance for understanding magnetic characteristics of many tissues. For this purpose, quantitative computational-based methods, implemented on top of the recognized clinical settings could be used as a robust and reproducible alternative to classical invasive techniques.

## Methods

Osirix DICOM viewer(Pixmeo, Switzerland) was used as the platform for developing and validating the proposed tool. Set of images with varying acquisition parameters - Inversion Time(TI) for T_1_ mapping and Echo Time(TE) for T_2_(*) mapping - were acquired. Linear least squared curve fitting was performed on a pixel-by-pixel basis and calculated values were saved in a separate file as the resulted map. For T_1_ mapping, -exp(-TI / T_1_) and for T_2_(*) mapping, -exp(-TE/T_2_) were used as time axis inputs in the linear fitting process. Coefficient of determination (*r*^2^) value was calculated for each pixel and used as a measure to evaluate the goodness of fit. To get perfusion maps, two relaxation time maps were used and perfusion values were calculated for each pixel using the formula: perf = λ * (1/map_1_ - 1/map_2_), in which map_1_ and map_2_ corresponds to values obtained for two relaxation time maps and λ is tissue-blood partition coefficient.

## Results

**Figure 1:**
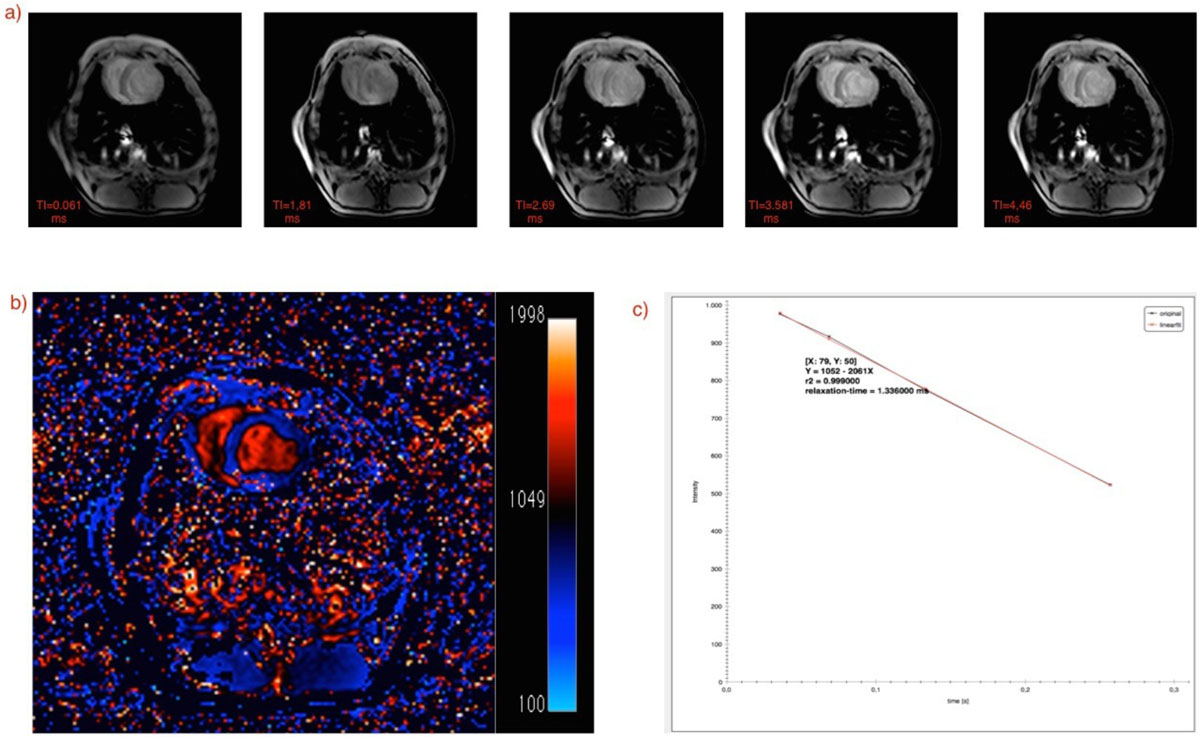
**An example of calculating parametric T**_**1**_ **relaxation time map a)input images with varying Inversion Times(TI) b) calculated T**_**1**_ **map c)plot demonstrating signal behavior and curved fit for a representative pixel located at [X:79, Y:50]**.

**Figure 2:**
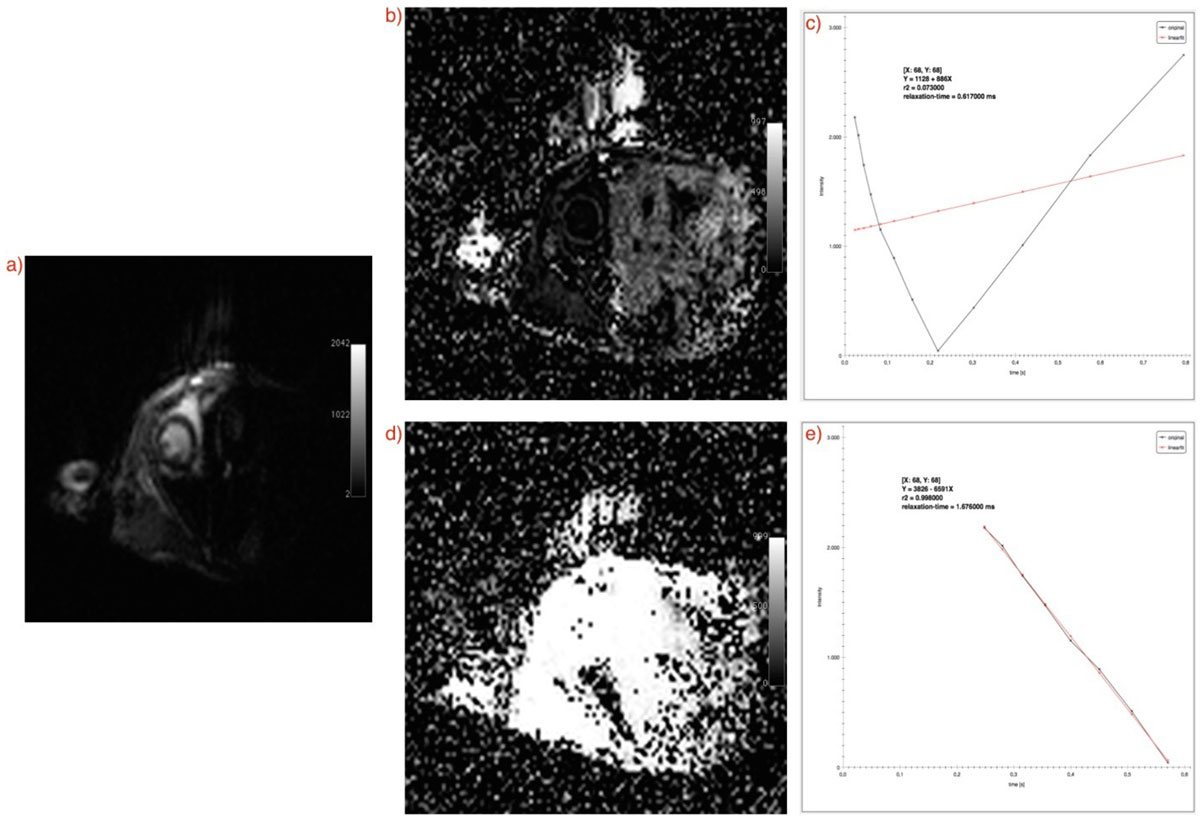
**Error reduction of linear curve fitting for T**_**1**_ **MOLLI mapping in a small animal study**. Input set has initially 12 images. Plots are shown for representative pixel located in position [X:68, Y:68] a)an example of input image b)coefficient of correlation map using 12 input images c)signal behavior plot demonstrates a poor linear fit due to the inconsistent signal behavior. d) 4 input images related to increasing slope of plot in c) have been eliminated. After rerunning the tool, coefficient of correlation map shows promising results for most of the pixels in regions of interest. e)signal behavoir plot for representative pixel confirms the good quality of linear fit

For validating the results, phantom, small animal and human data were used. Images were acquired using 7-Tesla MRI scanner from Varian® and 3-Tesla Philips Ingenuity TF Whole Body PET/MR Imaging System. Arterial Spin Labeling(ASL) and Modified Look-Locker Inversion Recovery(MOLLI) sequences for T_1_ and single-echo and multi-echo sequences for T_2_(*) were used. Obtained maps were analyzed using color-coded images. Furthermore, signal behavior was visualized for all coordinates(see Figure 1). By clicking on each single pixel, signal intensities for that location in the input series along with the information regarding the calculated fit(relaxation time, slope, intercept and goodness of fit) are shown. To reduce the error in fitting process, one or more images for each slice could be excluded from the calculations. This could be done after visualizing the fit for target pixels and detect images which increase the error(see Figure 2).

## Conclusions

Absolute quantitative relaxation time and perfusion maps for various MRI datasets were obtained using the developed open-source tool and on top of a well-known clinical setting. Pixel-based visualization of fitting process ensures end-users regarding the accuracy of the calculations. Finally, image processing features which has already been implemented in OsiriX will facilitate the visualization and processing of the generated maps.

